# Soil microarthropods respond differently to simulated drought in organic and conventional farming systems

**DOI:** 10.1002/ece3.7839

**Published:** 2021-06-28

**Authors:** Svenja Meyer, Dominika Kundel, Klaus Birkhofer, Andreas Fliessbach, Stefan Scheu

**Affiliations:** ^1^ Animal Ecology J.F. Blumenbach Institute for Zoology and Anthropology University of Göttingen Göttingen Germany; ^2^ Ecology Department of Biology University of Konstanz Konstanz Germany; ^3^ Department of Soil Sciences Research Institute of Organic Agriculture (FiBL) Frick Switzerland; ^4^ Department of Ecology Brandenburg University of Technology Cottbus Germany; ^5^ Centre of Biodiversity and Sustainable Land Use University of Göttingen Göttingen Germany

**Keywords:** Collembola, DOK trial, drought, organic farming, Oribatida, soil carbon

## Abstract

In Central Europe, summer droughts are increasing in frequency which threatens production and biodiversity in agroecosystems. The potential of different farming systems to mitigate detrimental drought effects on soil animals is largely unknown. We investigated the effects of simulated drought on the abundance and community composition of soil microarthropods (Collembola and Oribatida and Meso‐, Pro‐, and Astigmata) in winter wheat fields under long‐term conventional and organic farming in the DOK trial, Switzerland. We simulated drought by excluding 65% of the ambient precipitation during the wheat‐growing season from March to June 2017. The abundance of Collembola and Oribatida declined more consistently in conventionally managed fields compared to organically managed fields under simulated drought. The abundance of Collembola as well as Meso‐, Pro‐ and Astigmata, but not the abundance of Oribatida, increased in deeper soil layers due to simulated drought, suggesting vertical migration as a drought avoidance strategy. The species composition of Oribatida communities, but not of Collembola communities, differed significantly between drought treatments and between farming systems. Soil carbon content was a major factor structuring Oribatida communities. Our results suggest that organic farming buffers negative effects of drought on soil microarthropods, presumably due to higher soil carbon content and associated higher soil moisture and improved soil structure. This potential of organic farming systems to mitigate consequences of future droughts on soil biodiversity is promising and needs further exploration across larger climatic and spatial scales and should be extended to other groups of soil biota.

## Graphical abstract

Summer droughts are increasing in frequency which threatens production and biodiversity in agroecosystems. We investigated the potential of different farming systems to mitigate detrimental effects on soil fauna and found a more consistent decline in conventionally managed fields compared to organically managed fields. Our results suggest that this is due to higher soil carbon content, and associated higher soil moisture and improved soil structure, which allows vertical migration as response to drought.

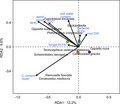

## 1 INTRODUCTION

Agriculture in Europe has experienced an intensification of management practices in the past decades, and agroecosystems are likely to be sensitive to the changing climate. Central Europe is facing changes in temperature as well as precipitation, and the magnitude of these changes is predicted to increase in the 21st century (EEA, 2017). Rising temperatures and a shift in precipitation toward the winter months increase the risk of summer droughts (Russo et al., 2013; Spinoni et al., 2015). Under these conditions, soil animals are likely to be more frequently exposed to reduced soil water content, which alters the availability of food resources (Bear et al., 2013) and the capacity to maintain homeostasis (Verhoef & Witteveen, 1980). Hence, crop plants will not only suffer from direct consequences of higher water stress in drought periods, but will also have to cope with changes in ecosystem functions that are provided by soil organisms (Kaneda & Kaneko, 2011; Yin, Eisenhauer, Auge, et al., 2019). Negative effects of drought conditions on soil organisms and crop plants might be mitigated by agricultural management practices that increase soil water‐holding capacity and provide additional resources. However, recent studies suggested that climate effects on soil fauna taxa vary little with land‐use intensity (Schädler et al., 2019; Yin, Eisenhauer, Schmidt, et al., 2019), but in particular, the abundance of Collembola may decrease under future climate conditions in organically, but not in conventionally, managed fields (Yin, Gruss, et al., 2019). Generally, however, combined effects of simulated drought and management practices on soil microarthropods in agroecosystems received little attention.

Soil microarthropods are adapted to more constant environmental conditions compared to aboveground arthropods. However, climate change also alters belowground conditions including temperature, CO_2_ levels, and water availability, with changes in precipitation presumably most severely affecting soil biota (Blankinship et al., 2011). Many soil organisms, from soft‐bodied springtails (Collembola) to heavily sclerotized oribatid mites (Oribatida), are known to be vulnerable to desiccation. Field experiments suggested that soil animals respond negatively to simulated drought (Blankinship et al., 2011; Petersen, 2011; Vestergård et al., 2015), but other studies did not report such effects (Kardol et al., 2011; Krab et al., 2014; Taylor et al., 2004). Yet, the majority of drought experiments has been performed in forests, which are more buffered against changes in abiotic conditions than open habitats, such as grasslands or arable fields. Soils in open habitats are generally more exposed to climatic conditions, and agricultural soils, in particular, are not well protected against extreme conditions during most parts of the year and therefore undergo pronounced annual fluctuations in soil moisture. These conditions may filter for species in agricultural soil animal communities that are generally adapted to drought conditions. However, these species may already live at the edge of their ecological niche in terms of climatic conditions and may not be able to tolerate even harsher conditions predicted for the future. Responses of soil animals to drought are likely to be taxon‐specific as there are variations in the individual drought tolerance and resilience of taxonomic groups (Lindberg & Bengtsson, 2005). Filtering of more drought‐tolerant species, therefore, would likely result in different and less diverse communities compared to less severe climatic conditions (Kardol et al., 2011; Makkonen et al., 2011; Petersen, 2011; Pflug & Wolters, 2001). An improved understanding of these filtering effects at species level provides the opportunity to identify indicator species for drought stress in soil communities.

Differences in biological and physicochemical soil properties between agricultural fields, even across geographical regions, are mainly driven by different management practices. In conventional farming systems, chemical pesticides and inorganic fertilizers are applied, whereas organic farming omits conventional pesticides and exclusively uses organic fertilizers, such as manure, compost, or slurry. The resulting higher levels of soil organic matter in organic farming systems (Gattinger et al., 2012) provide additional resources for decomposers, reflected in higher abundance of soil organisms in organically managed fields (Bengtsson et al., 2005; Birkhofer et al., 2008, 2012). High levels of organic matter cause structurally more complex soils and increase soil water‐holding capacity (Lotter et al., 2003) potentially mitigating negative effects of drought on soil animals. For a comprehensive understanding of future drought effects on biota in agroecosystems, it is therefore crucial to consider different farming systems and their potential to buffer against drought conditions.

The present study investigates the interactive effect of simulated drought and different long‐term farming systems on soil microarthropod communities. We compared microarthropod communities in conventionally and organically managed winter wheat fields in an agricultural long‐term experiment in Switzerland (DOK trial; Krause et al., 2020). Additionally, we experimentally manipulated soil moisture by establishing roofs that excluded 65% of the ambient precipitation. We hypothesized that (a) simulated drought reduces microarthropod abundances with these effects (b) being more pronounced under conventional management compared to organic management. We further hypothesized that (c) microarthropods migrate into deeper soil under simulated drought and that (d) individual species show specific responses to simulated drought resulting in different compositions of Collembola and Oribatida communities.

## 2 METHODS

### 2.1 Study site

**FIGURE 1:**
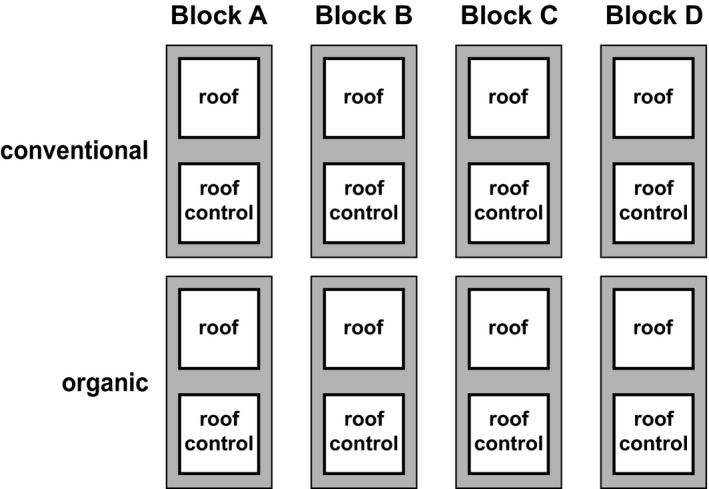
Scheme of the experimental design with four blocks (A–D) each containing one organic and one conventional field and each field with one control (roof control) and one drought treatment (roof)

The DOK trial is a long‐term experiment comparing organic and conventional agricultural management since 1978. It is located in Therwil in the Leimen Valley close to Basel, Switzerland (47°30′09.3″N, 7°32′21.5″E). Mean annual temperature is 10.5°C, and mean annual precipitation is 842 mm (Krause et al., 2020). The soil is a Haplic Luvisol (16% clay, 72% silt, 12% sand) on deposits of alluvial loess. For this study, we used winter wheat fields (*Triticum aestivum* L. cv. “Wiwa”). Eight experimental fields (each 5 × 20 m^2^) were located in four blocks, each including one organically (biodynamic) and one conventionally managed field (BIODYN and CONMIN treatments of the DOK trial, respectively; Figure 1). In each field, we established two types of roofs: one roof that excluded 65% of the ambient precipitation and a modified “control roof” that did not intercept rain, but controlled for potential artifacts caused by the roof construction itself. This results in a total number of eight replicates for the factors drought and farming system, respectively, and four replicates for the drought × farming system interaction for each sampling date. For details on the design of the experimental roofs, see Kundel et al. (2018). The roofs had a minimum distance from the field edges of 0.5 m. The organic farming system received only organic fertilizers (farmyard manure, compost, and slurry), and weeds were controlled mechanically. Further, biodynamic preparations were applied to soils, plants, and organic fertilizers (Krause et al., 2020; Kundel et al., 2020). In the organic farming system, twice during the experiment (March and April) 20 m^3^/ha slurry was applied. Fields in the conventional farming system received mineral fertilizer (40–60 kg N/ha in March, April, and May). Plant protection in the conventional farming system was carried out with insecticides, herbicides, and fungicides, according to threshold values as recommended by the producer (see Table S1 for details on pesticide products, amount of applied active ingredients, and application dates). Pesticides were applied with a knapsack‐sprayer with multiple nozzles. Additionally, plant growth regulators (1.5 L/ha Cycocel extra, Omya, in March) were applied in the conventional farming system. The experiment was established in March 2017 and lasted until shortly before harvesting at the end of June 2017. We sampled at four sampling dates: T0 in March before the roofs were established and T1‐T3 in April, May, and June, respectively.

### 2.2 Soil and plant properties

Soil samples were taken using a soil corer in the center of the plots to a depth of 20 cm, and the following soil properties were determined: water‐holding capacity, bulk density, pH, and concentrations of total phosphorus, phosphate, total organic carbon and total nitrogen (all at T0), and gravimetric soil water content (T0, T1, T2, T3), and mineral nitrogen (at T2). Additionally, plant properties (root dry weight, wheat biomass, wheat height, grain yield, weed cover, and concentrations of nitrogen and carbon of shoots and roots) and microbial activity (soil respiration) were measured (all at T2, except grain yield at T3). Further, data on the microbial community composition were obtained by measuring phospholipid fatty acids (PLFAs) and neutral lipid fatty acids (NLFAs) from soil samples at T2 (Kundel et al., 2020). We used the NLFA 16:1ω5 as measure of the amount of arbuscular mycorrhizal fungi (AMF) and converted it into biomass carbon using the following conversion factor: 1.047 nmol NLFA = 1 μg AMF biomass carbon (Olsson et al., 1995). For measuring the relative importance of nonmycorrhizal fungi and bacteria, we used the proportions of respective marker PLFAs to the total amount of PLFAs. The PLFAs i15:0, a15:0, 15:0, i16:0, 16:1ω9, i17:0, a17:0, cy17:0, 18:1ω7, and cy19:0 were used as markers for bacteria (Frostegård & Bååth, 1996) and the PLFA 18:2ω6 as marker for saprotrophic fungi (Olsson et al., 1995).

### 2.3 Soil animals

**FIGURE 2:**
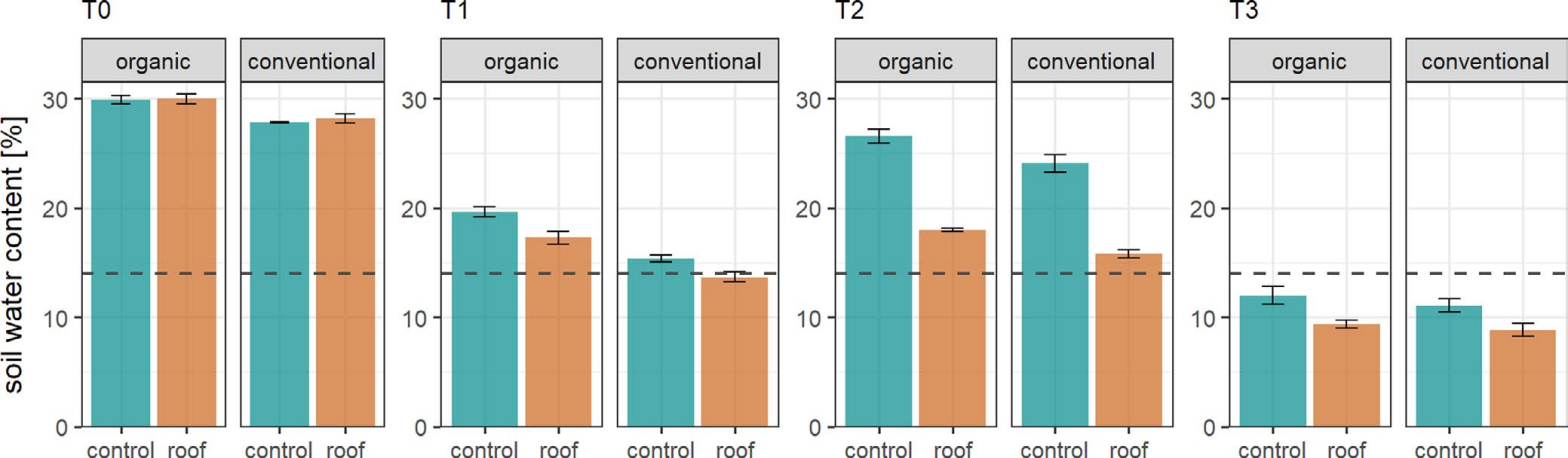
Gravimetric soil water content (0–20 cm depth) in control and drought treatments (roof) in wheat fields under organic and conventional management in March (T0, before roof establishment), April (T1), May (T2), and June (T3); dashed line, estimated wilting point; means ± *SE* based on four replicates

We took two soil cores, one of 5 and one of 20 cm diameter, under every roof at T1‐T3 covering a sampled area of 20 and 314 cm^2^, respectively, at each sampling time. Soil cores were taken to a depth of 10 cm and separated into upper (0–5 cm) and lower layer (5–10 cm). Animals were extracted by heat; temperature was gradually increased from 25 to 55°C over 10 days, for the large soil cores in steps of 5°C and for the small soil cores in steps of 2.5°C until 30°C and in steps of 5°C from 30 to 55°C per day (Kempson et al., 1963; Macfadyen, 1961). Animals were collected into a glycol–water solution (1:1), filtered, and stored in 70% ethanol. Animals were sorted to order level under a dissecting microscope (Stemi 2000; Zeiss). Additionally, we identified Collembola and Oribatida from the small soil cores of the second sampling campaign to species level using a microscope (Axioplan; Zeiss) and keys by Hopkin (2007), Fjellberg (1998, 2007), and Weigmann (2006). In addition, large Collembola (>1.5 mm) and Oribatida were identified from the large cores. We chose the second sampling campaign for species identification because differences in soil moisture were greatest at this sampling (see Figure 2).

### 2.4 Statistical analysis

Data were analyzed in R (R Development Core Team, 2020) using mixed‐effects models including field nested in block and drought nested in field (to account for multiple sampling dates) as random factors, and farming system and sampling date as fixed factors. The abundances of total Collembola, epigeic Collembola, and Oribatida and Meso‐, Pro‐, and Astigmata were analyzed using generalized linear mixed‐effects models (GLMMs) with a Poisson distribution using the R package *lme4* (Bates et al., 2015). The model for Meso‐, Pro‐, and Astigmata accounted for zero‐inflation by using the R package *glmmTMB* (Brooks et al., 2017). The model for Oribatida accounted for overdispersion using a negative binomial distribution. In the model for Collembola, we excluded data from the third sampling date due to excess zero‐count data. We analyzed differences in the depth distribution with linear mixed‐effects models (LMMs) using the same random effect structure as in the models for the abundance data. The depth distribution was expressed as the proportion of total individuals in the upper 0–5 cm of each sample; prior to the analyses, the data were arcsin square root transformed. Afterward, we run Wald chi‐square tests to inspect significances of the fixed effects. We only analyzed differences in depth distribution at the second sampling date when differences in soil moisture were most pronounced (Figure 2). We tested the fit of all GLMMs and LMMs with the function *testResiduals()* from the DHARMa package (Hartig, 2017).

Species richness of Collembola, epigeic Collembola, and Oribatida was analyzed using LMMs with field nested in block as random factors, and drought and farming system as fixed factors.

For the statistical analyses of taxonomic composition, all abundance data were log(*x* + 1) transformed to weigh down the importance of abundant species and a dummy variable (1) was added as recommended by Anderson et al. (2008). A Bray–Curtis resemblance matrix based on these data was then tested with permutational analyses of variance (PERMANOVA) with farming system and drought as fixed factors and 9,999 permutations of residuals under a reduced model. For significant model terms, similarity percentage analyses (SIMPER) were used to identify the most discriminating species (>25% individual contribution to Bray–Curtis dissimilarities). Nonmetric multidimensional scaling (NMDS) based in the same Bray–Curtis resemblance matrix was used to visualize the data. The PERMANOVA and SIMPER analysis were performed using the software PRIMER version 7.0.13 and the PERMANOVA add‐on (PRIMER‐e, Quest Research Limited, Auckland, New Zealand). Additionally, we used redundancy analysis (RDA) to evaluate interrelationships between the measured soil, plant and microbial parameters, and the community composition of total Collembola, epigeic Collembola, and Oribatida. All constraining factors were standardized to a range between 0 and 1 to account for different scales of the variables included. We used the function *ordistep()* for model selection with a stepwise addition of constrains to the null model based on the AIC selection criteria using permutation tests. From the full set of the measured variables, weed cover, water‐holding capacity, and carbon content of the roots were identified as the most relevant factors. We then added variables related to drought and farming system (TOC, water content) as well as potential resources for Oribatida and Collembola (AMF, proportion of bacterial and fungal PLFAs, and root dry weight) as explanatory variables to the model. From this model, we excluded AMF biomass because it was highly correlated with TOC. We assessed the significance of these factors by ANOVA‐like permutation tests using the function *anova*.*cca()*. For the RDA, we used the vegan package in R (Oksanen et al., 2019). See Table S2 for all predictor variables included in the RDA.

## 3 RESULTS

### 3.1 Soil water content

Soil water content was consistently lower in the drought than in the control treatment (Figure 2). However, the effect of simulated drought varied with time and was most pronounced at T2 (significant drought × sampling date interaction; *χ* ^2^ = 224.1, *p* < 0.001). Moreover, soil water content was higher in organically managed fields than in conventionally managed fields at all sampling dates except for T3. At T3, the soil water content generally was very low irrespective of the farming system with <10% in the drought and <13% in the control treatments, both being below the estimated wilting point of 14%.

### 3.2 Abundance of soil animals

**FIGURE 3:**
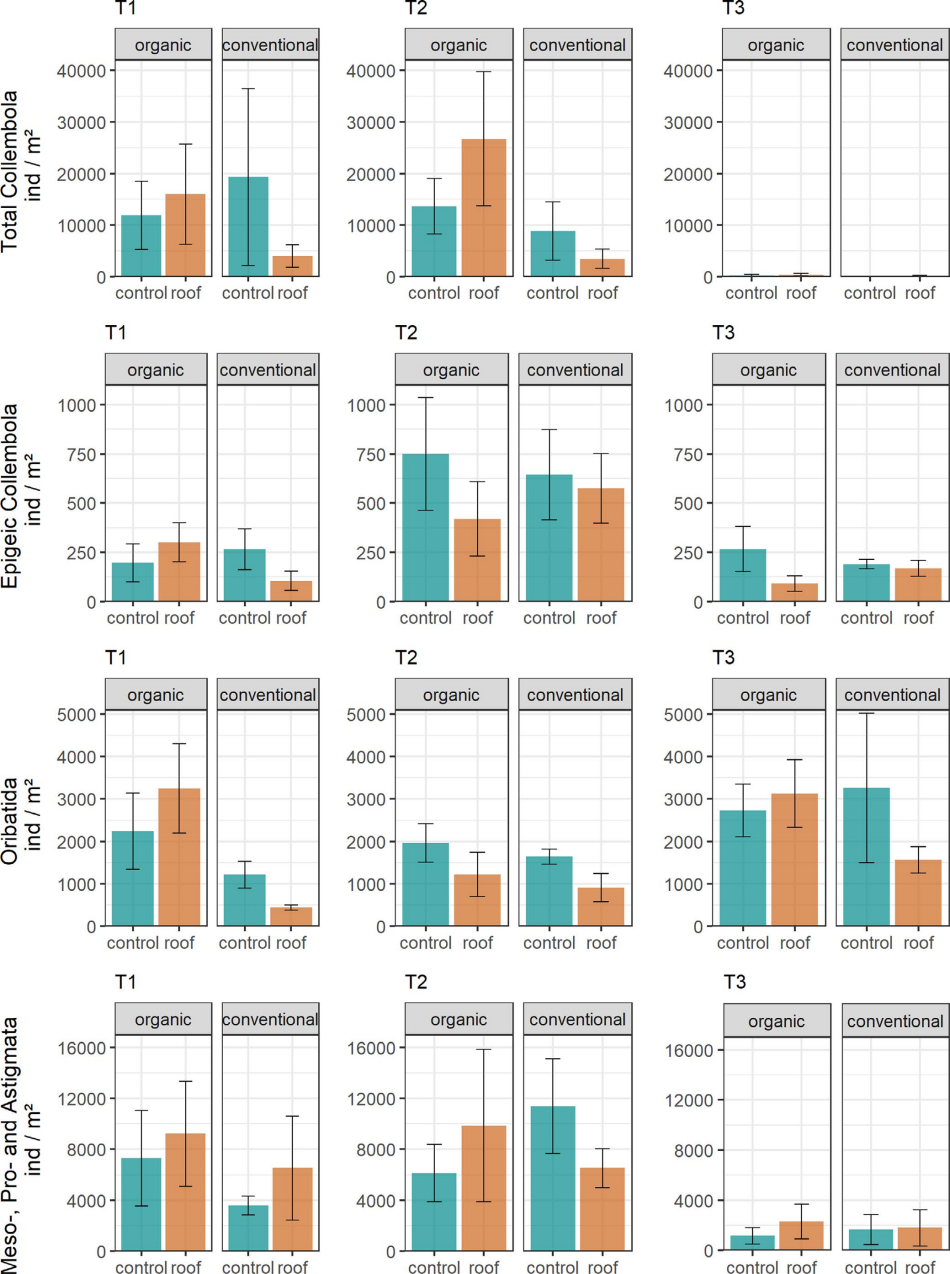
Abundance of total Collembola, epigeic Collembola, and Oribatida and Meso‐, Pro‐, and Astigmata in control and drought treatments (roof) under organic and conventional management in April (T1), May (T2), and June (T3). Abundances are given in individuals per square meter; means ± *SE* based on four replicates; for statistical analyses, see Table 1

The effect of simulated drought on mesofauna abundances differed between the two farming systems, and this interaction differed among animal groups (Figure 3; Table 1). Drought reduced the abundance of Collembola and Oribatida in conventionally, but not in organically, managed fields. On the contrary, drought reduced the abundance of epigeic Collembola in organically, but not in conventionally, managed fields at T2 and T3. By contrast, drought increased the abundance of Meso‐, Pro‐, and Astigmata at T1 in both farming systems, whereas at T2 it was higher under drought in organically but lower in conventionally managed fields.

**TABLE 1:** Results (*χ* ^2^‐values) of the GLMM (type III sum of squares) on the effect of drought, farming system, and sampling date on the abundance of total Collembola, epigeic Collembola, and Oribatida and Meso‐, Pro‐, and Astigmata

	Total Collembola			Epigeic Collembola			Oribatida			Meso‐, Pro‐, and Astigmata		
	*χ* ^2^	*df*	*p*‐value	*χ* ^2^	*df*	*p*‐value	*χ* ^2^	*df*	*p*‐value	*χ* ^2^	*df*	*p*‐value
Drought (D)	0.119	1,21	0.73	3.083	1,33	0.079	1.427	1,33	0.232	0.041	1,33	0.839
Farming system (FS)	9.116	1,21	**0.003**	11.452	1,33	**<0.001**	24.624	1,33	**<0.001**	0.636	1,33	0.425
Sampling date (S)	20.794	1,21	**<0.001**	25.318	2,33	**<0.001**	9.033	2,33	**0.011**	13.835	2,33	**<0.001**
D × FS	6.602	1,21	**0.01**	10.640	1,33	**0.001**	7.122	1,33	**0.008**	1.558	1,33	0.212
D × S	4.284	1,21	**0.039**	17.402	2,33	**<0.001**	3.221	2,33	0.2	8.335	2,33	**0.016**
FS × S	5.288	1,21	**0.022**	18.715	2,33	**<0.001**	11.573	2,33	**0.003**	1.539	2,33	0.463
D × FS × S	0.606	1,21	0.436	20.690	2,33	**<0.001**	3.099	2,33	0.212	24.809	2,33	**<0.001**

Note

Significant *p*‐values are given in bold.

### 3.3 Depth distribution of soil animals

**FIGURE 4:**
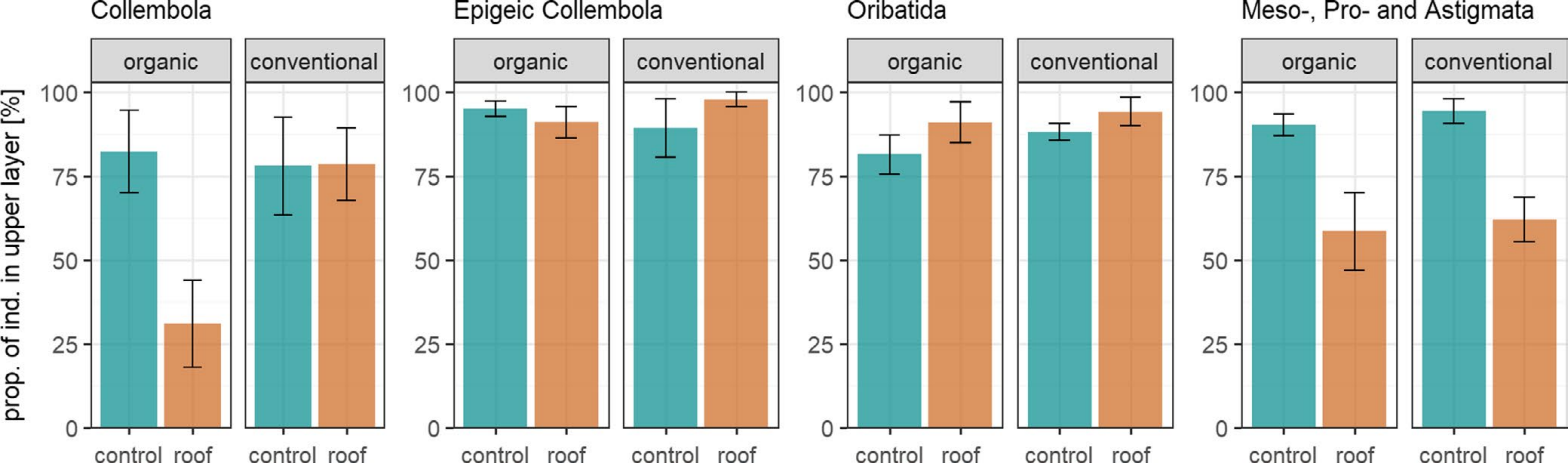
Depth distribution of Collembola, epigeic Collembola, and Oribatida and Meso‐, Pro‐, and Astigmata in control and drought treatments (roof) under organic and conventional management as proportion of individuals (of total) in the upper layer (0–5 cm); means ± *SE* based on four replicates

Collembola as well as Meso‐, Pro‐, and Astigmata migrated to deeper soil (5–10 cm) in the drought treatment, whereas epigeic Collembola were not affected and Oribatida even showed the opposite pattern (Figure 4). In Collembola, however, movement into deeper soil was restricted to the organically managed fields (marginally significant drought × farming system interaction, *χ* ^2^ = 2.7, *p* = 0.098). In Meso‐, Pro‐, and Astigmata, movement into deeper soil was consistent in both farming systems (Meso‐, Pro‐, and Astigmata: *χ* ^2^ = 24.1, *p* < 0.001). On the contrary, Oribatida moved into the upper soil layer (0–5 cm) in the drought treatment in both farming systems (*χ* ^2^ = 5.7, *p* = 0.017).

### 3.4 Species composition

**FIGURE 5:**
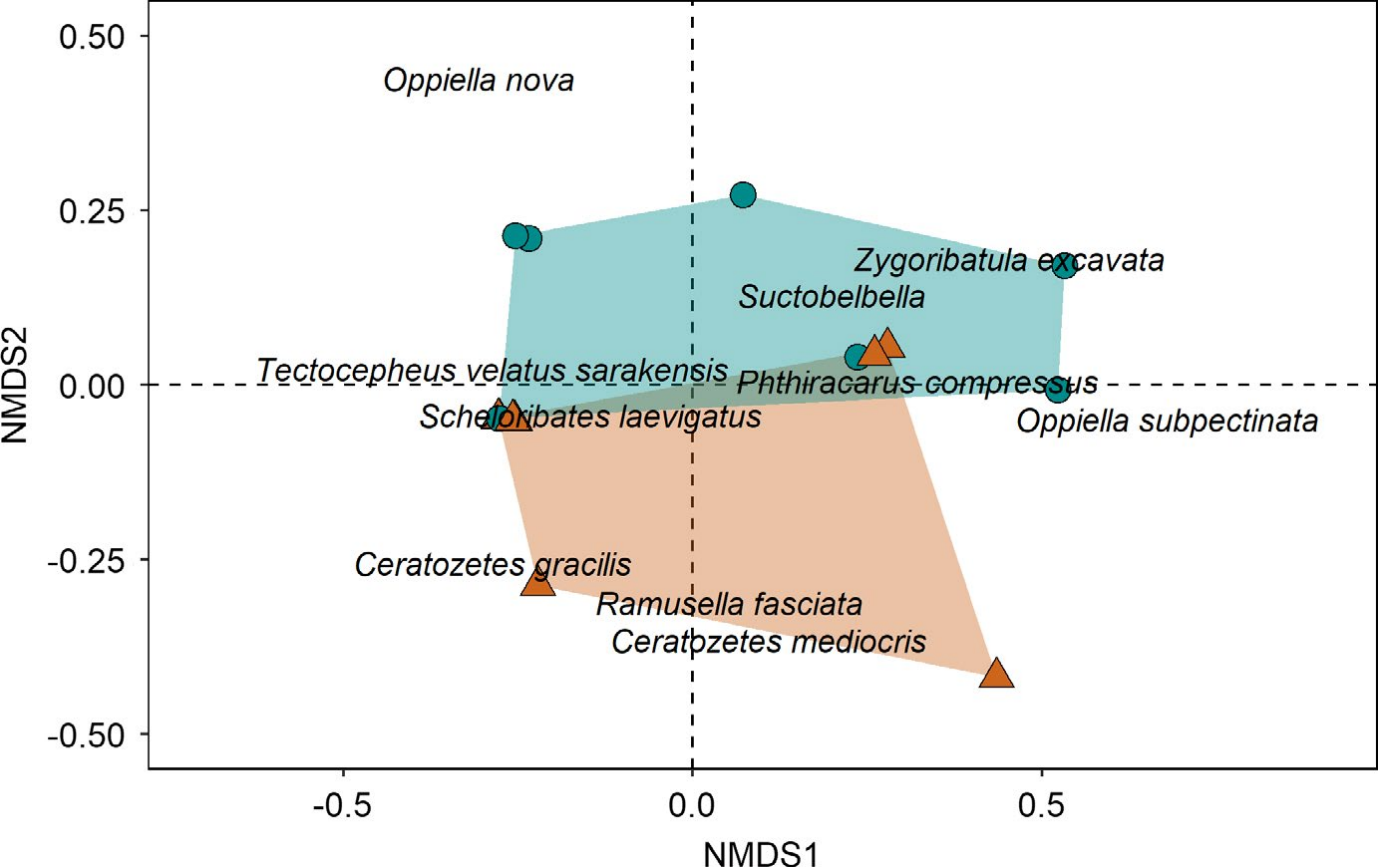
NMDS ordination based on the Oribatida community composition in control and drought treatments as reflected by the first and second NMDS dimensions. Colored polygons frame sites of the control (turquoise; circle) and drought treatment (orange; triangle)

**FIGURE 6:**
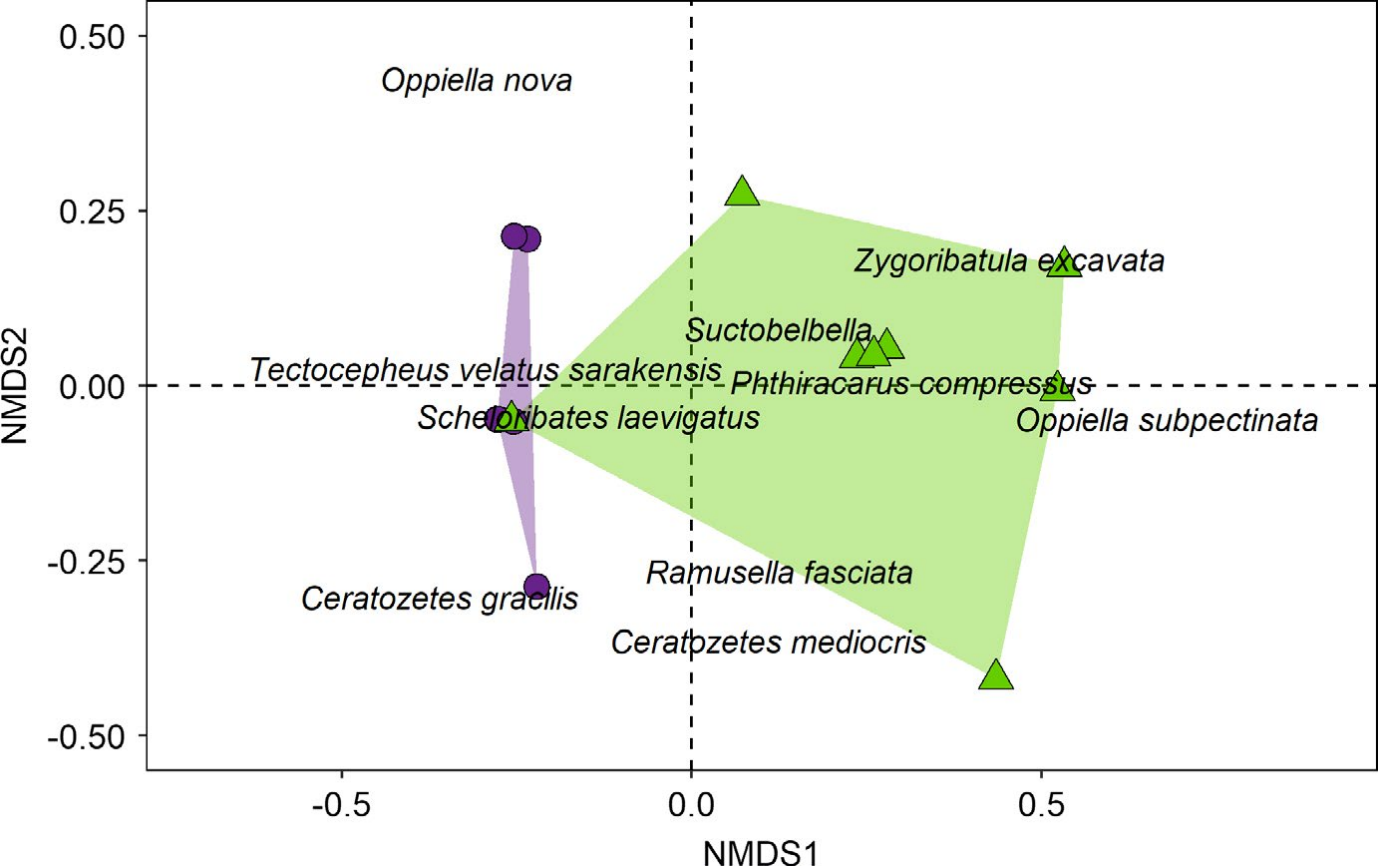
Oribatida community composition in organic and conventional farming as reflected by the first and second NMDS dimensions. Colored polygons frame sites of organic (green; triangle) and conventional farming (violet; circle)

**FIGURE 7:**
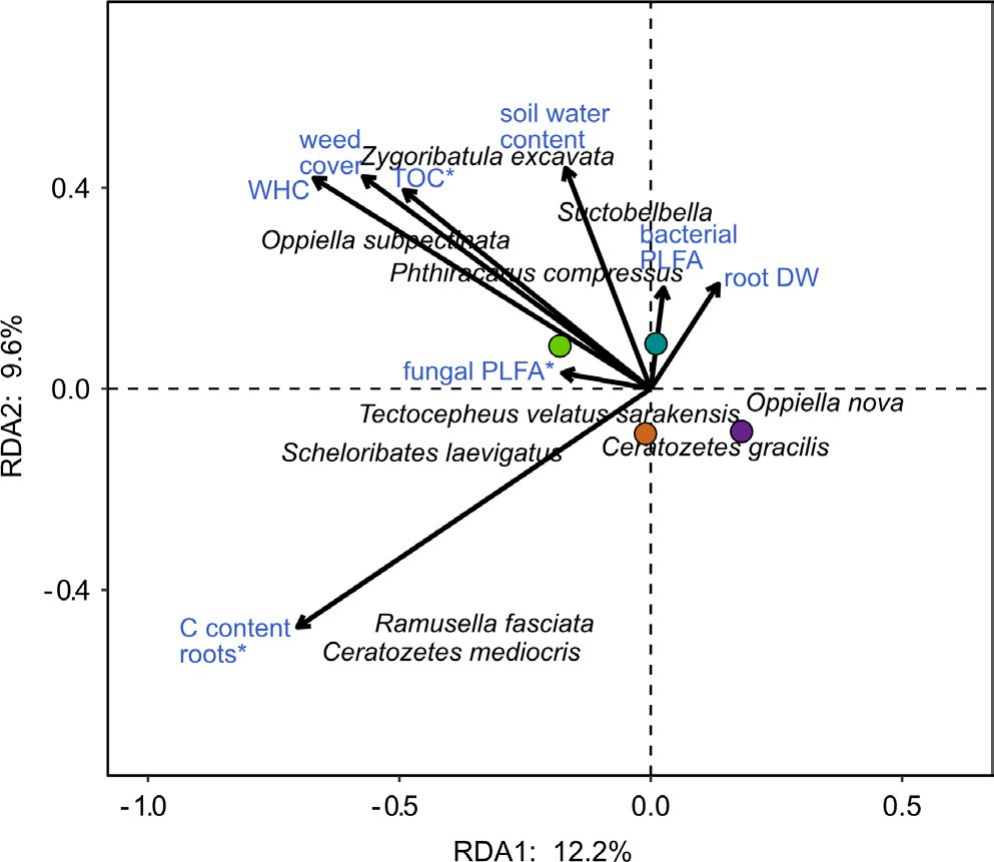
Species–environment relationships of Oribatida in two farming systems (organic, green, and conventional, purple) and two drought treatments (roof control, turquoise, and roof, orange) as indicated by RDA ordination. Environmental variables included carbon (C) content of roots, weed cover, water‐holding capacity (WHC), total organic carbon (TOC), soil water content, proportion of bacterial and fungal phospholipid fatty acids (PLFA), and root dry weight (DW). Significant environmental variables are marked with asterisks

Species richness of Oribatida was significantly higher in organically managed fields than in conventionally managed fields (*F*_1,3_ = 12.78, *p* = 0.037), but did not differ between the drought treatments. Species richness of Collembola was reduced by simulated drought in organically managed fields (drought × farming system interaction; *F*_1,6_ = 7.71, *p* = 0.032). Species richness of epigeic Collembola did not differ significantly between the farming systems and between the drought treatments. The NMDS ordination (stress = 0.057) separated Oribatida communities in the drought treatment from communities in the control (Figure 5). Also, the NMDS ordination separated the Oribatida communities of the two farming systems (Figure 6). Supporting these separations, drought (*F*_1,12_ = 2.40, *p* = 0.046) and farming system (*F*_1,12_ = 11.20, *p* = 0.002) were significant in the respective model, with no significant interaction term. The significant differences between farming systems were mainly due to *Oppiella subpectinata* (SIMPER: 29.2% contribution to dissimilarity between farming systems) and *Zygoribatula excavata* (26.2%) being exclusively present in the organically, but missing from the conventionally, managed fields. No single Oribatida species contributed more than 25% to the dissimilarity between drought treatments. Contrary to Oribatida, neither drought nor farming system significantly affected the community composition of epigeic and total Collembola. Species–environment relationships were only significant in Oribatida with the first and second axes explaining 12.2% and 10% of the variation in species composition, respectively (adjusted *R* ^2^ = 0.331; Figure 7). Significant predictors were total organic carbon (*F*_1,7_ = 2.37, *p* = 0.011), proportion of fungal PLFAs (*F*_1,7_ = 2.63, *p* = 0.007), and carbon content of roots (*F*_1,7_ = 4.14, *p* = 0.005), with total organic carbon being closely related to the organic farming system. In line with the NMDS results, the species *Zygoribatula excavata* and *Oppiella subpectinata*, but also *Phthiracarus compressus,* were associated with the organic system.

## 4 DISCUSSION

### 4.1 Abundance

Collembola and Oribatida were most sensitive to drought, but as hypothesized, the decline in abundance was more severe in conventionally managed fields than in organically managed fields. This indicates that negative effects of drought are mitigated by conditions in the organic farming system. A major difference between the two farming systems is the fertilization with compost, manure, and slurry in the organic system and synthetic, inorganic NPK fertilizer in the conventional system. This difference results in a higher concentration of soil organic matter in the organic system, which is positively correlated with water‐holding capacity of soils (Shepherd et al., 2002). Indeed, soil water content was generally higher in organically managed fields compared to conventionally managed fields when soil moisture was above the estimated wilting point.

Organic farming systems further are known to have higher microbial biomass and activity compared to conventional systems (Birkhofer et al., 2012). For the current field experiment, Kundel et al. (2020) reported higher fungal and bacterial abundances and microbial respiration in the organically managed fields compared to the conventionally managed fields. Microbes serve as an important food resource for many soil microarthropods (Chahartaghi et al., 2005; Schneider et al., 2004), and their availability may alter effects of simulated drought on microarthropod abundances. At our field site, drought increased Collembola abundances in organically managed fields and decreased them in conventionally managed fields. Though microbial biomass was higher in the organically managed fields, microorganisms might be in part inaccessible for Collembola when soil moisture is too high. Microarthropods move in air‐filled spaces through the soil, and movement might be hindered when soil pores are filled with water (Schimel, 2018). Under simulated drought, lower soil moisture therefore may have increased the accessibility of microorganisms as food and thereby resulted in increased abundance of Collembola in organically managed fields. These effects on Collembola may have cascaded up to higher trophic levels as Meso‐, Pro‐, and Astigmata partly followed the abundance responses of Collembola, which are potential prey for Meso‐ and Prostigmata (Koehler, 1999). Oribatida abundance also decreased under simulated drought in the conventional system at all sampling dates. However, the abundance of Oribatida did not increase under simulated drought in the organically managed fields suggesting that, in contrast to Collembola, Oribatida did not benefit from higher resource availability. Contrasting to our second hypothesis, the abundance of epigeic Collembola decreased with drought mainly in organically managed fields. Epigeic Collembola may have benefitted from the presence of an herb layer, which was absent in the conventionally managed fields. Herbaceous plants may provide both habitat and food resources for epigeic Collembola (Potapov et al., 2016), and negative effects of drought on herbs thereby may have detrimentally affected epigeic Collembola. Though simulated drought did not affect the percentage cover of herbs in organically managed fields, plant water stress may have detrimentally affected epigeic Collembola as shown for herbivores (Huberty & Denno, 2004).

### 4.2 Vertical distribution

Drought effects on soil microarthropods might be mitigated by improved soil structure with larger pores in organically managed fields allowing vertical movement to avoid drier upper soil layers. Supporting our third hypothesis, drought increased Collembola abundance in deeper soil layers, but only in organically managed fields, where high amounts of organic matter may support a more structured soil (Shepherd et al., 2002). In fact, in the DOK trial, Mäder et al. (2002) found soil aggregate stability to be 10%–60% higher in organically managed fields compared to conventionally managed fields. Parallel to the higher abundance of Collembola in deeper soil layers, total Collembola abundance was higher under drought only in organically managed fields at the second sampling date. Exploitation of additional resources in deeper soil layers may have contributed to this abundance pattern. In contrast to total Collembola, epigeic Collembola, mainly colonizing the soil surface, were not found in the deeper soil layers under simulated drought, and consistent with this, their abundance also declined in the well‐structured soil of the organically managed fields at T2 and T3.

In contrast to our third hypothesis, Oribatida did not move into deeper soil layers under simulated drought, indicating that, compared to Collembola, they did not benefit from the improved soil structure in the organic farming system. Consequently, their abundance decreased under drought simulation in both farming systems. Similar to our study, Perdue and Crossley (1990) also found that most mites did not migrate to deeper soil layers, even when abundances declined dramatically during periods of low soil moisture in agricultural fields. However, in our study, Meso‐, Pro‐, and Astigmata followed in part the depth distribution of Collembola. Their relative abundance in the upper soil layer decreased under drought, although less strongly than in Collembola. However, in contrast to Collembola, Meso‐, Pro‐, and Astigmata migrated to deeper soil in both organically and conventionally managed fields suggesting that these taxonomic groups can better cope with the less structured soils in the conventional system.

### 4.3 Temporal changes

Toward the end of the experiment, ambient drought conditions decreased soil water content dramatically to an average of 10.3% of dry weight, that is, below the estimated wilting point of 14%. At this very low level, the small remaining differences in soil water content between the drought and control treatment probably were of little relevance for soil microarthropod communities. The generally very low abundance of soil mesofauna at the last sampling date, therefore, presumably was due to an overall low soil moisture overriding roof effects of the previous sampling dates. The changes in abundance during the three sampling dates suggest different population dynamics for each microarthropod group as response to naturally occurring changes in soil moisture. While the abundance of total Collembola and epigeic Collembola and Meso‐, Pro‐, and Astigmata peaked at T1 and T2, and decreased severely at T3, the abundance of Oribatida peaked at T3. Highest abundance of Oribatida at T3 indicates that they are not only able to survive, but even able to thrive under low moisture conditions in arable fields, probably due to low metabolic rates and slow development (Norton, 1994). However, simulated drought reduced the abundance increase of Oribatida from T2 to T3 in the conventionally managed fields, indicating that low abundances early in the season (T1 and T2) could not be compensated toward T3.

Collembola, on the other hand, reproduce fast allowing the buildup of high population densities early in the season (T1 and T2), which then dramatically collapsed at T3 when soil moisture levels were very low. This suggests that Collembola are generally more sensitive to drought than Oribatida. However, fast reproduction also enables fast recolonization and this likely contributes to the fast recovery of Collembola populations after disturbances in agricultural fields. In fact, Alvarez et al. (1999) found that watering of arable fields after a 4‐month drought period provoked immediate hatching from eggs in several Collembola species. Furthermore, Collembola are known to recolonize previously hostile habitats faster than Oribatida by wind drift and active locomotion (Dunger et al., 2002; Lehmitz et al., 2011). The abundance dynamics of Meso‐, Pro‐, and Astigmata at the three sampling dates resembled that of Collembola again indicating that Meso‐ and Prostigmata were trophically linked to Collembola. The similar response of Collembola and Astigmata may be due to the fact that both taxa are little sclerotized (contrasting to Oribatida), rendering similar sensitivity to drought.

### 4.4 Community composition

Overall, species richness of Collembola and Oribatida was rather low; however, in Oribatida, it was generally higher in organically managed fields than conventionally managed fields irrespective of simulated drought, whereas in Collembola total species richness was reduced by simulated drought but only in organically managed fields. Again, this suggests higher sensitivity of Collembola than Oribatida to drought. By contrast, the species structure of Oribatida, but not that of Collembola communities, reflected the drought treatments. Previous studies on drought effects from other nonforest, open habitats (mainly grasslands) reported changes (Kardol et al., 2011; Lindberg et al., 2002; Pflug & Wolters, 2001; Yin, Gruss, et al., 2019) or no changes (Holmstrup et al., 2013; Krab et al., 2014) of Collembola community composition, but rarely included Oribatida. Generally, Oribatida are perceived as being poor bioindicators, because they only respond slowly to changes in environmental conditions due to their long life cycles (Behan‐Pelletier, 1999). Contrasting this assumption and our fourth hypothesis, Oribatida communities differed significantly between the drought and control treatments, although no individual species was characteristic for a specific treatment and Oribatida communities were relatively species‐poor. Notably, effects already occurred 3 months after the start of drought simulation.

Oribatida community composition also differed between the two farming systems, whereas again, this was not the case for Collembola communities. Differences in the community structure of Oribatida between the farming systems were mainly due to *Zygoribatula excavata* and *Oppiella subpectinata*, which were significantly more abundant in organically managed fields compared to conventionally managed fields. Both species are known from forest and grassland habitats with high amounts of soil organic matter (Weigmann, 2006). Our study suggests that these species also colonize agricultural fields, in particular farming systems with high levels of soil carbon. Soil carbon content was an important driver for Oribatida communities in our study sites favoring *Z. excavata* and *O. subpectinata*. A significant effect of soil carbon on mite communities also has been found in previous studies (Minor & Norton, 2004; Scheu & Schulz, 1996; Wissuwa et al., 2013). The relative abundance of fungi also affected Oribatida community composition in our study. Fungi form a major part of the diet of Oribatida, including species of the family Oribatulidae and Scheloribatidae, such as *Z. excavata* and *Scheloribates laevigatus* (Schneider et al., 2004), abundant at our study sites. Moreover, the carbon content of roots significantly affected the species composition of Oribatida possibly via rhizodeposition, feeding on dead roots or root‐associated fungi (Pollierer et al., 2007).

Although the different farming systems in the DOK trial have been established more than 40 years ago, Collembola communities did not differ significantly between the systems, which is consistent with previous studies (Alvarez et al., 1999; Birkhofer et al., 2008). Our results showed that the abundance of Collembola may dramatically decrease in cereal fields at the end of the growing season and this likely increases the risk of extinction of local populations. It needs to be studied whether these responses are associated with drought conditions and whether they are aggravated by water uptake of crop plants. Agricultural practices such as tillage, but in particular drought events, may prevent the establishment of stable Collembola communities in future agroecosystems.

## 5 CONCLUSIONS

Our findings show that the vulnerability of soil microarthropods against drought in agricultural fields depends on the farming system with more severe negative impacts of drought in long‐term conventional farming systems compared to organic farming systems. The results suggest that soil carbon content is among the most important factors driving differences between farming systems and indicate that soils with high carbon content may buffer detrimental effects of future drought conditions on soil animal communities. The observed beneficial effects of high soil carbon content in this study likely were driven by higher soil moisture and improved soil structure under organic farming. Improved soil structure may promote the ability of soil microarthropods to migrate vertically, thereby allowing them to avoid most severe drought conditions in the upper soil layers. Community responses to simulated drought as well as community differences between the farming systems were found for Oribatida but not for Collembola. This indicates that Oribatida communities respond to both short‐term (drought) and long‐term (farming system) changes in environmental conditions. The community composition of Oribatida, rather than that of Collembola, therefore may serve as indicators for effects of drought and management on soil biota.

## CONFLICT OF INTEREST

The authors declare that they have no conflict of interest.

## AUTHOR CONTRIBUTION

**Svenja Meyer:** Data curation (lead); Formal analysis (lead); Investigation (lead); Methodology (lead); Writing‐original draft (lead). **Dominika Kundel:** Conceptualization (equal); Data curation (equal); Methodology (equal); Project administration (equal); Resources (equal); Writing‐review & editing (equal). **Klaus Birkhofer:** Conceptualization (lead); Formal analysis (equal); Funding acquisition (lead); Methodology (lead); Project administration (equal); Resources (equal); Writing‐review & editing (equal). **Andreas Fliessbach:** Conceptualization (equal); Funding acquisition (equal); Methodology (equal); Resources (lead); Writing‐review & editing (equal). **Stefan Scheu:** Conceptualization (lead); Formal analysis (equal); Funding acquisition (lead); Methodology (equal); Resources (equal); Supervision (lead); Writing‐review & editing (lead).

## Supporting information

Table S1‐S2

## Data Availability

Mesofauna abundance data: Dryad https://doi.org/10.5061/dryad.80gb5mkr3.
