# Supplementary material for: Soil microarthropods respond differently to simulated drought in organic and conventional farming systems
Source: Ecol Evol. 2021 Jun 28;11(15):10369–80. doi: 10.1002/ece3.7839 (PMC8328414; doi:10.1002/ece3.7839)
Supplement: Supplementary file 1 — Table S1‐S2 [file ECE3-11-10369-s001.docx]

**Appendix**

S1 Pesticide application performed in the conventional farming system

| **Date of application** | **Type of pesticide** | **Product** | **Manufacturer** | **Active ingredients** | **Applied product (l/ha)** | **Applied active ingredients (g/ha)** |
| --- | --- | --- | --- | --- | --- | --- |
| March, 20 | herbicide | HusarOD (Bayer, Zollighofen) | Bayer (Zollighofen) | Iodosulfuron-methyl-natrium | 0.1 | 10 |
|  |  |  |  | Mefenpyr-diethyl (Safener) |  | 30 |
| March, 20 | herbicide | Mondera | Syngenta (Switzerland) | Diflufenican | 1 | 33.3 |
|  |  |  |  | MCPP-P |  | 500 |
| April, 11 | fungicide | Pronto plus | Bayer (Zollighofen) | Sprioxamin | 1.5 | 375 |
|  |  |  |  | Tebuconazol |  | 200 |
| May, 30 | fungicide | Aviator Xpro | Bayer (Zollighofen) | Bixafen | 1 | 75 |
|  |  |  |  | Prothioconazol |  | 150 |
| May, 30 | fungicide | Miros FL | Bayer (Zollighofen) | Chlorothalonil | 1 | 500 |
| May, 30 | insecticide | Audienz | Omya (Oftringen) | Spinosad | 0.1 | 48 |

**S2** Soil (water holding capacity, total organic carbon, water content), plant (root dry weight, carbon content of roots, weed cover) and microbial parameters (proportion of fungal and bacterial PLFAs) in the different farming systems (conventional, organic) and drought treatments (control, roof) used in the RDA (see Fig. 6).

| **Farming system** | **Drought** | **Water holding capacity (%)** | **Total organic carbon (%)** | **Soil water content (%)** | **Root dry weight (g/l of soil)** | **Total weed cover (%)** | **Root carbon content (%)** | **Proportion of fungal PLFAs (%)** | **Proportion of bacterial PLFAs (%)** |
| --- | --- | --- | --- | --- | --- | --- | --- | --- | --- |
| Conventional | Control | 41.93 | 1.45 | 26.27 | 0.63 | 0.00 | 39.78 | 1.37 | 44.22 |
|  |  | 38.50 | 1.12 | 23.03 | 0.60 | 0.00 | 41.41 | 1.49 | 46.10 |
|  |  | 38.86 | 1.25 | 24.47 | 0.65 | 0.00 | 40.30 | 1.36 | 43.41 |
|  |  | 34.41 | 1.29 | 22.59 | 0.70 | 0.00 | 38.06 | 1.32 | 47.37 |
|  | Roof | 42.48 | 1.44 | 15.66 | 0.76 | 0.00 | 39.20 | 1.53 | 43.63 |
|  |  | 39.52 | 1.11 | 14.89 | 1.12 | 0.00 | 37.33 | 1.72 | 45.20 |
|  |  | 39.76 | 1.21 | 16.67 | 0.78 | 0.00 | 37.63 | 1.63 | 44.99 |
|  |  | 37.81 | 1.25 | 16.07 | 0.85 | 0.00 | 38.73 | 1.41 | 45.92 |
| Organic | Control | 45.55 | 1.63 | 28.22 | 0.64 | 15.00 | 40.43 | 1.51 | 45.26 |
|  |  | 39.81 | 1.50 | 26.46 | 0.91 | 70.00 | 42.04 | 1.30 | 47.24 |
|  |  | 46.47 | 1.59 | 26.36 | 0.90 | 35.00 | 40.48 | 1.61 | 45.33 |
|  |  | 38.11 | 1.67 | 25.15 | 0.59 | 30.00 | 42.94 | 1.36 | 46.75 |
|  | Roof | 46.08 | 1.64 | 17.59 | 0.45 | 55.00 | 41.22 | 1.44 | 43.99 |
|  |  | 41.61 | 1.66 | 18.39 | 0.72 | 15.00 | 38.89 | 1.52 | 46.07 |
|  |  | 39.86 | 1.45 | 17.98 | 0.61 | 35.00 | 50.54 | 1.41 | 45.51 |
|  |  | 38.71 | 1.68 | 18.09 | 0.69 | 40.00 | 40.45 | 1.44 | 44.36 |
